# Valve-in-Valve Repair in a Critically Ill Obstetric Patient with Severe Pulmonary Stenosis: A Rare Case

**DOI:** 10.3390/healthcare13121361

**Published:** 2025-06-06

**Authors:** Alixandria F. Pfeiffer, Hadley Young, Oxana Zarudskaya, Nora Doyle, Syed A. A. Rizvi

**Affiliations:** 1 Department of Obstetrics and Gynecology, Division of Maternal Fetal Medicine, University of Texas Health San Antonio, San Antonio, TX 78229, USAzarudskaya@uthscsa.edu (O.Z.);; 2 College of Biomedical Sciences, Larkin University, Miami, FL 33169, USA; srizvi@larkin.edu

**Keywords:** tetralogy of Fallot, pulmonic valve stenosis, valve-in-valve repair, cardio-obstetrics, critical care obstetrics, congenital heart disease

## Abstract

**Background:** Among patients with congenital heart disease, particularly those with a history of undergoing the Fontan operation, pregnancy presents a significant maternal–fetal risk, especially when complicated by severe valvular dysfunction. Lung reperfusion syndrome (LRS) is a rare but life-threatening complication occurring following valve intervention. Multidisciplinary management, including by Cardio-Obstetrics teams, is essential for optimizing outcomes in such high-risk cases. **Methods**: We present the case of a 37-year-old pregnant patient with previously repaired tetralogy of Fallot (via the Fontan procedure) who presented at 24 weeks gestation with worsening severe pulmonary stenosis and right-ventricular dysfunction. The patient had been lost to cardiac follow-up for over a decade. She experienced recurrent arrhythmias, including supraventricular and non-sustained ventricular tachycardia, prompting hospital admission. A multidisciplinary team recommended transcatheter pulmonic valve replacement (TPVR), performed at 28 weeks’ gestation. **Results**: Post-TPVR, the patient developed acute hypoxia and hypotension, consistent with Lung Reperfusion Syndrome, necessitating intensive cardiopulmonary support. Despite initial stabilization, progressive maternal respiratory failure and fetal compromise led to an emergent cesarean delivery. The neonate’s neonatal intensive care unit (NICU) course was complicated by spontaneous intestinal perforation, while the mother required intensive care unit (ICU)-level care and a bronchoscopy due to new pulmonary findings. She was extubated and discharged in stable condition on postoperative day five. **Conclusions**: This case underscores the complexity of managing severe congenital heart disease and valve pathology during pregnancy. Lung reperfusion syndrome should be recognized as a potential complication following TPVR, particularly in pregnant patients with Fontan physiology. Early involvement of a multidisciplinary Cardio-Obstetrics team and structured peripartum planning are critical to improving both maternal and neonatal outcomes.

## 1. Introduction

Pulmonary valve stenosis is a rare but serious condition that poses significant risks for obstetric patients, particularly those who are critically ill [1]. The physiological adaptations of pregnancy, including increased cardiac output, blood volume, and heart rate, can intensify preexisting cardiovascular conditions, increasing the likelihood of maternal and fetal complications [2]. Severe pulmonary stenosis, which impedes blood flow from the right ventricle to the pulmonary artery, can lead to right-ventricular failure, arrhythmias, and hypoxemia, making timely intervention essential [3]. Traditional surgical valve replacement, though effective, carries substantial risks for pregnant patients due to the physiological vulnerabilities associated with pregnancy. Valve-in-Valve (ViV) repair has emerged as a promising, minimally invasive alternative that offers reduced recovery times and lower perioperative risks [4]. This approach is particularly advantageous for high-risk obstetric patients, as it helps stabilize cardiac function while minimizing procedural complications, emphasizing the need for a multidisciplinary approach in managing these complex cases [5].

The introduction of transcatheter ViV techniques, originally developed for aortic valve disease, provides a viable treatment option for high-risk populations, including those with severe pulmonary stenosis. While ViV transcatheter aortic valve replacement (TAVR) has been shown to lead to reduced perioperative mortality compared to redo surgical replacement, its application in pulmonary valve disease, especially in pregnant patients, remains underexplored [6]. Pregnancy places additional hemodynamic stress on the cardiovascular system, increasing the risk of decompensation in patients with pulmonary stenosis. ViV repair offers a means of avoiding high-risk open-heart surgery, reducing procedural time, and limiting the requirement for a cardiopulmonary bypass, a feature that is particularly beneficial for obstetric patients with reduced physiological reserves [7,8]. However, challenges such as prosthesis–patient mismatch, potential coronary obstructions, and the need for precise imaging-guided placement must be addressed [9]. In this case report, we have highlighted the potential of ViV repair in the management of a critically ill obstetric patient with severe pulmonary stenosis, emphasizing the utility of and advocating for a multidisciplinary approach involving cardiologists, maternal–fetal medicine specialists, and critical care teams to optimize outcomes.

## 2. Case Presentation

A 37-year-old G2P0111 with a history of congenital heart disease (CHD) including a previously repaired tetralogy of Fallot (TOF) and Fontan physiology presented at 24 weeks’ gestation to our Level IV tertiary care center for cardiac evaluation. She had been lost to cardiology follow-up for over a decade. An outside echocardiogram revealed severe pulmonic valve (PV) stenosis, a right-ventricular systolic pressure (RVSP) of 120–130 mmHg, severe tricuspid regurgitation, and moderate-to-severe right-ventricular (RV) enlargement with impaired systolic function. The patient was admitted for continuous telemetry monitoring and experienced recurrent supraventricular tachycardia (SVT) and non-sustained ventricular tachycardia (NSVT) between 24 and 28 weeks’ gestation.

Given the escalating arrhythmias and concern for electrical instability, the multidisciplinary care team (maternal–fetal medicine/cardiology/congenital cardiology/neonatal intensive care unit/extracorporeal membrane oxygenation/cardiothoracic surgery/cardiothoracic anesthesia/obstetric anesthesia) determined that antiarrhythmic therapy alone would be insufficient. She was maintained on metoprolol succinate (50 mg daily). Due to the high risk of ventricular arrhythmia, TPVR was recommended to reduce the arrhythmic burden and improve hemodynamics.

Her prior surgical history included a left-sided Blalock–Taussig (BT) shunt at 3 days of age, TOF repair with a pulmonary homograft at age 3, and revision to a 25 mm bovine pericardial valve at age 15. At 28 weeks’ gestation, she underwent successful TPVR with a 26 mm Edwards SAPIEN valve. While initially stable post-extubation, she developed acute hypoxic respiratory failure and hypotension within 3.5 h.

**Figure 1:**
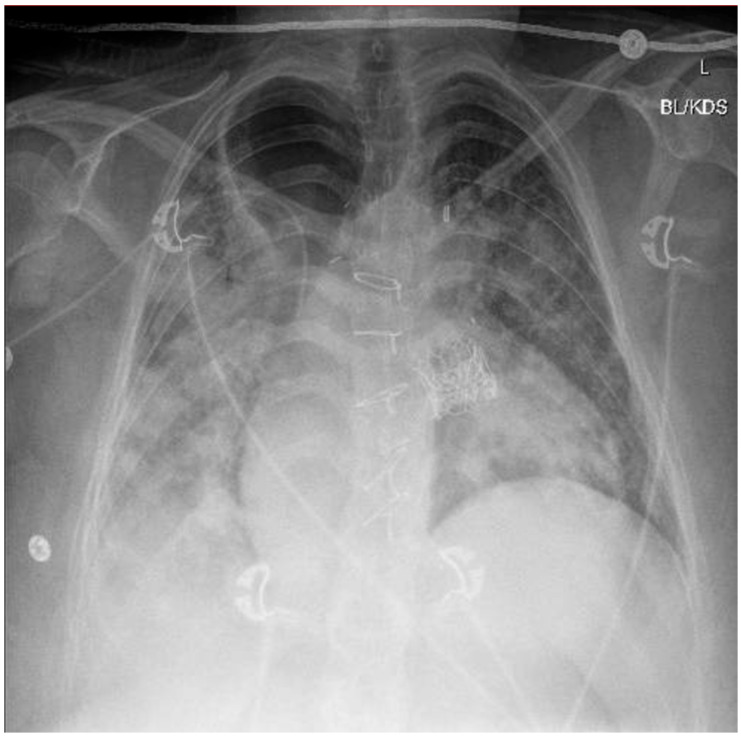
Postop chest X-ray (CXR) revealing increased multifocal airspace opacities most prominent in the right lung with a mediastinal shift and diffuse bilateral reticular opacities indicative of Lung Reperfusion Syndrome. Additionally median sternotomy wires from previous coronary artery bypass grafting (CABG) and TAVR can be noted.

A chest X-ray and point-of-care ultrasound revealed diffuse bilateral airspace opacities and B-lines without consolidation, consistent with flash pulmonary edema (Figure 1). LRS—an uncommon complication characterized by non-cardiogenic pulmonary edema following sudden restoration of forward flow—was strongly suspected. Echocardiography excluded pulmonary embolism and right heart failure. Bronchoscopy revealed dense mucoid and sanguineous clots in the right upper lobe without purulence. Despite concern for aspiration, an infectious etiology was considered unlikely based on negative cultures, the lack of a fever, and rapid resolution of leukocytosis (initial WBC 27 × 10^9^/L).

Management included high-flow nasal cannula (HFNC) oxygen administration, intravenous diuretics (later changed to PO), norepinephrine vasopressor support, and empiric piperacillin–tazobactam administration, which was discontinued after five days. The patient remained on IV pantoprazole (40 mg BID) throughout her hospitalization.

Due to worsening maternal hypoxia and signs of fetal distress, she underwent emergent cesarean delivery at 28 weeks and 2 days’ gestation. Post-delivery, the patient was extubated, weaned off oxygen, and discharged home on postoperative day 5 with 2 L of nasal cannula oxygen, a dual antiplatelet therapy (DAPT) course, and metoprolol (25 mg). Discharge medications included acetaminophen, aspirin, docusate, famotidine, ferrous sulfate, hydrocortisone, ibuprofen, lanolin ointment, magnesium hydroxide and oxide, pantoprazole, prenatal vitamins, and simethicone. During the two-month cardiology follow-up, cardiac function remained stable. She was advised to continue taking aspirin throughout the course of her life and Plavix for six months, with a follow-up echocardiogram planned to take place after four months.

The neonate, born weighing 960 g, required initial intubation for respiratory distress and was re-intubated on day of life 2 for spontaneous intestinal perforation. Following an 83-day NICU course, the infant was discharged home, where the infant was fed using a nasogastric tube, and is still being monitored at our institution’s PREMIEre Clinic.

## 3. Discussion

This case underscores the complexity and risks associated with TPVR during pregnancy, particularly regarding the rare but serious complication posed by LRS. TPVR was pursued for our patient due to severe pulmonic stenosis, tricuspid regurgitation, and progressive arrhythmias. Antiarrhythmic therapy was not trialed; instead, after extensive multidisciplinary case discussions, the escalating frequency of arrhythmias was interpreted as a marker of electrical instability, warranting urgent intervention to reduce maternal morbidity and fetal risk.

Approximately 3.5 h post-TPVR, the patient developed acute hypoxia and hypotension. A chest X-ray and point-of-care ultrasound revealed bilateral airspace opacities and B-lines without consolidation—findings consistent with flash pulmonary edema. LRS, a rare but serious complication resulting from the abrupt restoration of forward flow through a previously obstructed or stenotic valve, was strongly suspected. The underlying pathophysiology involves capillary leakage, oxidative stress, and alveolar flooding [10,11]. Echocardiography excluded pulmonary embolism and right heart failure. Bronchoscopy showed mucoid and sanguineous clots without purulence, and all cultures remained negative. Although aspiration was initially considered, especially given the patient’s history of achalasia, the lack of fever and rapid clinical improvement with diuresis made this less likely.

Management included prompt diuresis, vasopressor support, broad-spectrum antibiotics (piperacillin-tazobactam), and high-flow nasal cannula (HFNC) oxygen administration. Despite initial leukocytosis (WBC 27 × 10^9^/L), infection was ultimately excluded. Electrolytes were closely monitored, and furosemide administration was transitioned from IV to oral. The patient remained on IV pantoprazole (40 mg BID) throughout hospitalization. She was discharged on postoperative day five with 2 L of nasal cannula oxygen, dual antiplatelet therapy (DAPT), and metoprolol. Two-month follow-up showed stable cardiac function, and she was prescribed a course of aspirin (to be taken throughout the course of her life) and a planned six-month course of Plavix.

This case also highlights significant fetal risks. The neonate, born at 28w2d, weighing 960 g, required intubation for respiratory distress and again on day of life 2 for spontaneous intestinal perforation. After an 83-day NICU course, the infant was discharged home, where the child was to be fed using nasogastric tubes, and is still being taken for multidisciplinary follow-ups.

As shown in the prospective cohort study by Wichert-Schmitt et al. [12], pregnant patients with right-sided bioprosthetic valves (BPVs) face maternal cardiac and fetal adverse event rates of approximately 11% and 21%, respectively. In our patient, BPV dysfunction led to persistent arrhythmias and ultimately necessitated TPVR, which was followed by maternal LRS and preterm delivery. This case demonstrates how BPV-related deterioration in pregnancy can trigger a cascade of events impacting both maternal and fetal outcomes.

The timing of cardiac intervention in the case of pregnancy remains a complex decision. A meta-analysis by van Steenberg et al. [13] found no association between maternal mortality and trimester at the time of cardiac surgery. However, the same study noted significantly higher fetal mortality when cesarean delivery (CS) occurred after cardiac surgery. Accordingly, our multidisciplinary team determined that if CS was feasible and safe, delivery should be prioritized before intervention to mitigate adverse fetal outcomes.

LRS, though rare, presents unique challenges in the obstetric population. The physiologic changes of pregnancy, including increased plasma volume, elevated cardiac output, and reduced systemic vascular resistance, may amplify the severity of LRS and prolong maternal recovery [12,14]. In our case, LRS manifested as abrupt pulmonary edema and hemodynamic collapse, requiring urgent postpartum support and delivery.

Anticipating this complication requires vigilant monitoring during the early post-procedural period—particularly within the first 24 h. Early recognition and coordinated management are essential, with readiness to initiate respiratory support, administer diuretics, manage hemodynamic instability, and mobilize rapid delivery plans if fetal compromise ensues being paramount. A dedicated Cardio-Obstetrics team is vital in executing this comprehensive response.

Finally, this case reflects the broader challenge posed by the limited pregnancy-specific data on TPVR. Current guidelines are extrapolated from nonpregnant populations [4,15]. Ethical and safety concerns have led to the exclusion of pregnant individuals from interventional trials, resulting in a critical evidence gap [14,16,17,18]. Consequently, management decisions are often based on expert consensus and small case series [19,20]. Our case contributes to the evolving literature by documenting a rare but serious complication and reinforces the urgent need for prospective registries and standardized protocols in order to guide maternal–fetal care in the case of high-risk cardiovascular pregnancies [21].

## 4. Conclusions

Pulmonary valve stenosis in critically ill obstetric patients presents significant management challenges due to the physiological adaptations of pregnancy, which exacerbate underlying cardiovascular conditions. Traditional surgical interventions carry substantial risks, making minimally invasive techniques such as ViV TPVR a compelling alternative. This case demonstrates the successful use of TPVR in a pregnant patient with severe pulmonary stenosis and Fontan physiology, underscoring the importance of employing a collaborative, multidisciplinary approach in order to optimize maternal and fetal outcomes. Of particular concern is the potential for LRS, a rare but serious complication occurring following TPVR. LRS can occur rapidly and present with acute hypoxic respiratory failure, severe pulmonary edema, and hemodynamic instability. Vigilant post-procedural monitoring and preparedness for emergent respiratory support are essential. This case reinforces the pivotal role of coordinated Cardio-Obstetrics teams, including maternal–fetal medicine, cardiology, cardiothoracic surgery, critical care, and anesthesia departments, in anticipating and managing such complications.

ViV TPVR offers a promising, less invasive strategy for stabilizing cardiac function while minimizing perioperative risks. However, success relies heavily on timely referral, careful patient selection, structured procedural planning, and comprehensive post-intervention monitoring.

Looking ahead, we propose the following directions to advance care in this field:

1. Conduct prospective evaluations of TPVR and other transcatheter valve therapies in relation to pregnancy, ideally through multicenter registries that can systematically capture maternal–fetal outcomes and procedural complications.
2. Develop clinical protocols to identify obstetric patients at high risk for LRS, particularly those with Fontan physiology or longstanding bioprosthetic valve dysfunction.
3. Create standardized multidisciplinary peripartum planning algorithms for patients undergoing high-risk cardiac interventions during pregnancy.

Continued research is urgently needed to refine best practices, guide the safe timing of cardiac procedures, and improve outcomes for both mothers and their children. The integration of maternal–fetal medicine and cardiovascular disciplines remains fundamental in managing congenital and structural heart disease during pregnancy.

## Data Availability

Data sharing is not applicable to this article as no datasets were generated or analyzed during the current study.
